# Successful Volume Control of Invasive Pituitary Adenoma Tumor With Pasireotide: A New Horizon for a Challenging Disease

**DOI:** 10.1210/jcemcr/luaf075

**Published:** 2025-04-18

**Authors:** Tarik Elhadd, Shahd I Ibrahim, Elabbass Abdelmahmuod, Susan M Webb

**Affiliations:** Pituitary Clinic, Endocrine Section, Department of Medicine, Hamad General Hospital, Hamad Medical Corporation, P.O. Box 3050, Doha, Qatar; Pituitary Clinic, Endocrine Section, Department of Medicine, Hamad General Hospital, Hamad Medical Corporation, P.O. Box 3050, Doha, Qatar; Pituitary Clinic, Endocrine Section, Department of Medicine, Hamad General Hospital, Hamad Medical Corporation, P.O. Box 3050, Doha, Qatar; Department of Medicine, Univ Autonoma Barcelona, Research Center for Pituitary Diseases, Institut de Recerca Sant Pau (IIB-Sant Pau) and CIBERER Unit 747, ISCIII, Department of Endocrinology, Hospital S Pau, Barcelona 08025, Spain

**Keywords:** pituitary tumor, nonfunctioning pituitary tumor, resistant invasive pituitary adenoma, pasireotide

## Abstract

A 62-year-old Sudanese female was diagnosed with a nonfunctioning pituitary adenoma in 2006. Despite undergoing 3 transsphenoidal surgeries and radiotherapy, her tumor persisted, causing debilitating symptoms, including headaches and visual defects. As a last resort, she was treated with pasireotide, a second-generation somatostatin analog with a broader receptor affinity than first-generation drugs. Over 6 months, the tumor mass was stabilized, and her symptoms, including headaches, disappeared. This case demonstrates a potential new therapeutic possibility for treating resistant pituitary adenoma with pasireotide, offering hope for patients when traditional treatments fail.

## Introduction

Pituitary adenomas are the third most frequently diagnosed intracranial tumors, with nonfunctioning adenomas accounting for 14% to 54% of all pituitary adenomas [1, 2]. Nonfunctioning adenomas are usually benign tumors that do not cause hormone oversecretion, except for hyperprolactinemia secondary to pituitary stalk compression, which occurs in around 40% of cases [3]. They are typically identified incidentally on imaging or from symptoms of mass effects, such as headache, vision changes, or apoplexy. First-line treatment is transsphenoidal surgery (TSS); however, because of frequent suprasellar or parasellar extensions, total resection is often not possible and tumor regrowth or recurrence is common [1, 4]. Persistent or recurrent adenomas are usually treated with radiation therapy. In a small proportion of these cases, drug treatment with dopamine agonists and, to a lesser extent, somatostatin analogs (SSAs) may achieve reduction or stabilization of the tumor [5].

Previous studies have shown that somatostatin receptor subtypes (SSTRs) are expressed in pituitary adenomas, with different subtypes expressed differently in nonfunctioning pituitary adenomas. Consequently, octreotide long-acting release (LAR) has been assessed for efficacy in these tumors [6]. Given the broader spectrum of binding affinity of pasireotide to SSTR subtypes, pasireotide LAR has been previously trialed in nonfunctioning pituitary adenoma. These preliminary studies showed that pasireotide can achieve tumor stabilization, with improvement in associated symptoms [7, 8]. Thus, there is evidence for a potential use of pasireotide in patients with aggressive nonfunctioning pituitary adenoma resistant to standard treatments. The following case report describes the benefits of using pasireotide in a patient with a long-standing and particularly difficult-to-treat pituitary adenoma.

## Case Presentation

A 62-year-old Sudanese woman presented with multiple comorbidities, including type-2 diabetes mellitus, hypertension, coronary artery disease, chronic obstructive pulmonary disease, and glaucoma. She had received platelet-rich plasma injections for bilateral osteoarthritis of the knees. In 2006, she was diagnosed with a very invasive nonfunctioning pituitary adenoma and underwent TSS the following year in Saudi Arabia. In 2008, she underwent radiotherapy. In 2018, she had a second TSS in Qatar, despite which magnetic resonance imaging (MRI) in September 2021 showed persistent adenomatous tissue, measuring 16 × 14.5 × 13 mm.

**Figure 1.**
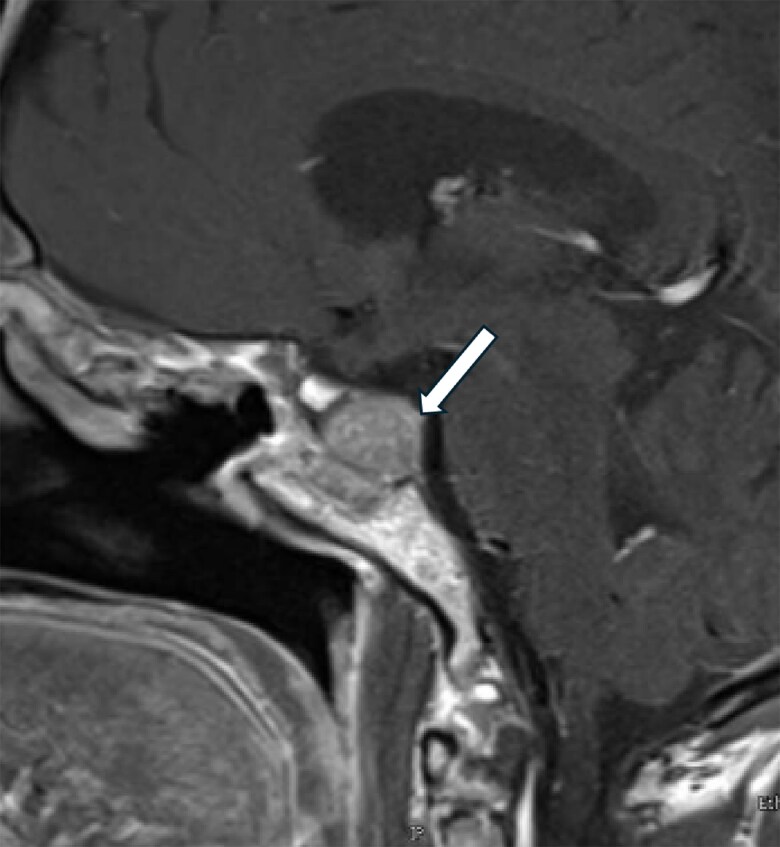
Residual pituitary adenoma measures 17.3 × 15.7 × 13.2 mm in anteroposterior, craniocaudal, and transverse diameters, respectively (October 2022).

**Figure 2.**
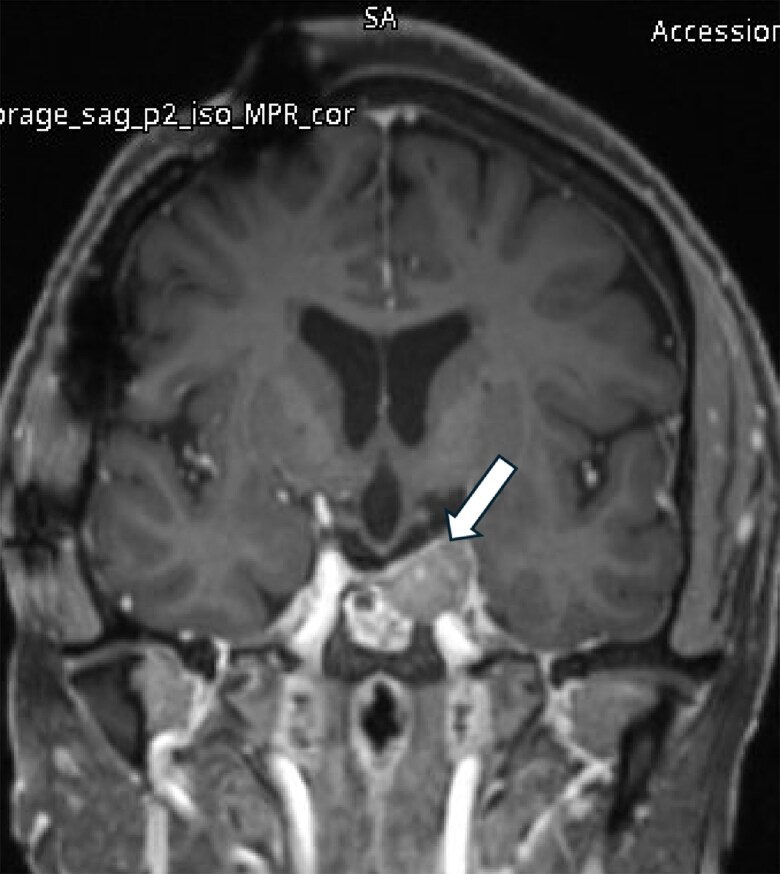
Residual pituitary adenoma measures 22.1 × 18.7 × 15.8 mm in its maximum anteroposterior, craniocaudal, and transverse diameters, respectively (May 2023).

She underwent a third transsphenoidal surgery in Qatar in October 2022. Subsequently, she developed panhypopituitarism, which was managed with levothyroxine 125 μg and hydrocortisone 20 mg in the morning and 10 mg in the afternoon. She experienced temporal hemianopia in the right eye and a superior arcuate defect in the left eye. A pituitary MRI in October 2022 revealed an increase in the size of the residual pituitary tissue in the left posterolateral aspect of the sella, measuring 17.3 × 15.7 × 13.2 mm in anteroposterior, transverse, and craniocaudal dimensions (Fig. 1). A follow-up MRI in May 2023 showed that the tumor continued to grow, measuring 22.1 × 18.7 × 15.8 mm. It was abutting the left cavernous sinus with no evidence of invasion of the left internal carotid artery (Fig. 2). The patient reported frequent headaches. These were not lateralized but centrally positioned, right in the top of the skull vault. They were of migrainous type, persisting for 2 days or more and associated with nausea but no vomiting.

While pasireotide has primarily been used for the treatment of acromegaly and Cushing disease, there is also some evidence suggesting it may be effective in nonfunctioning pituitary adenoma, including tumors that may be compressing surrounding structures or causing symptoms due to their size [9-11]. Pasireotide at a dose of 40 mg intramuscular every month was thus initiated. The patient reported initial side effects in the form of mild abdominal discomfort, which later resolved, but chose to pursue the treatment. Her hemoglobin A1c (HbA1c; normal range 4-5.6%) worsened from 8.4% to >12% 3 months after initiation of the medication (the patient had been diagnosed with type 2 diabetes in 2008 and was on vildagliptin/metformin 50/1000 mg twice daily in addition to insulin aspart 20 U 3 times daily and insulin glargine 20 units once daily; after worsening of her HbA1C, her insulin requirements increased by 50%). After daily headaches for the previous 7 months, the patient reported immediate relief after starting pasireotide.

She lost weight, from 89 to 76 kg, and reported an improved general sense of well-being and greater physical fitness, with more energy and better sleep with a resulting improvement in her daily functioning and perceived daily quality of life (no formal quality of life questionnaire was used).

**Figure 3.**
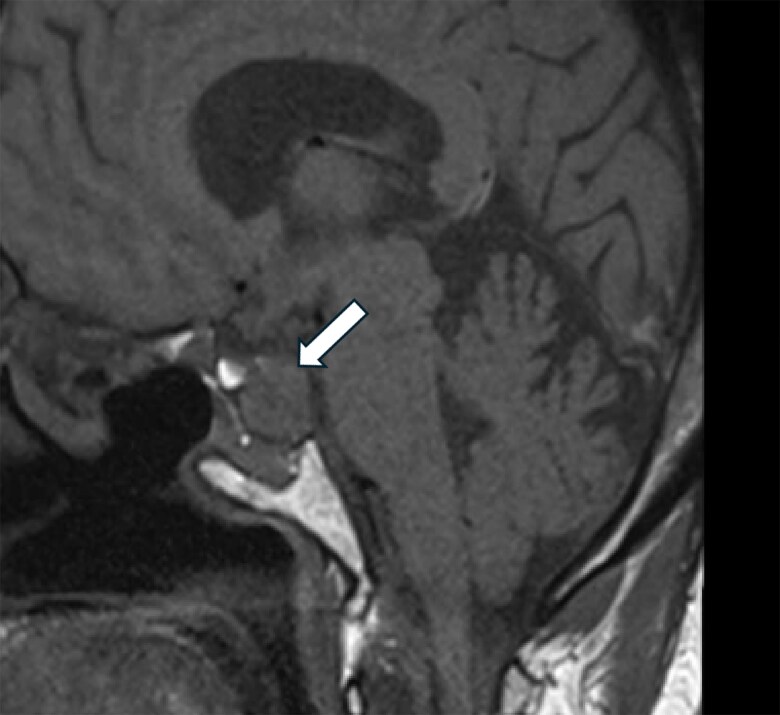
Residual pituitary adenoma measures 22.1 × 18.3 × 15.4 mm in anterior-posterior, transverse, and craniocaudal dimensions (January 2024).

**Figure 4.**
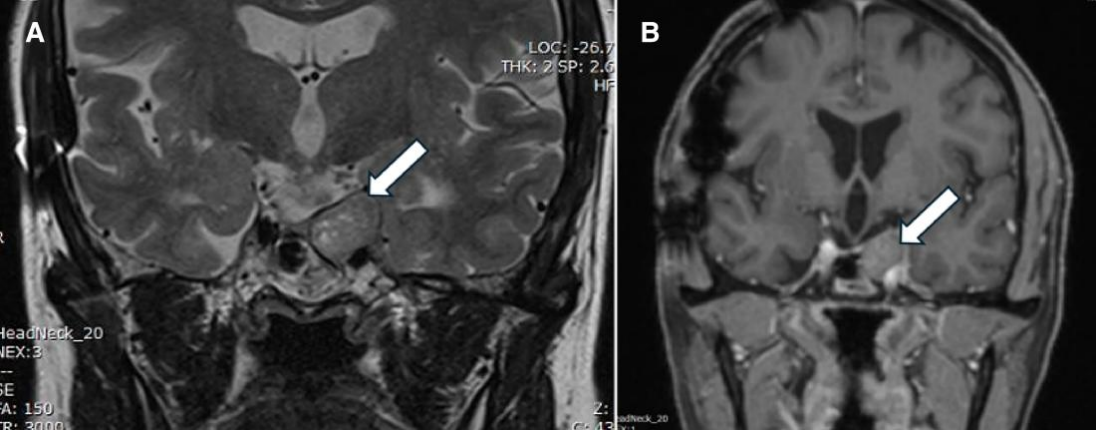
(A) Precontrast and (B) postcontrast images of the patient following treatment with pasireotide (May 2024).

After completing 1 year of therapy in July 2024, she continued to report an improved sense of well-being at a dose of 60 mg of pasireotide intramuscular/month with a stabilization of the tumor mass, which had not grown further (MRI in January 2024 showed that the tumor measured 22.1 × 18.3 × 15.4 mm; Fig. 3). Coronal images before and after the provision of pasireotide are shown in Fig. 4.

## Diagnostic Assessment

The patient's diagnosis of nonfunctioning pituitary adenoma was confirmed by MRI; clinical symptoms (headache, right temporal hemianopia, and left superior arcuate defects); endocrine evaluation that revealed panhypopituitarism and required full hormonal replacement therapy with levothyroxine and hydrocortisone; and pathology that showed tumor cells positive for synaptophysin and chromogranin and negative for ACTH, prolactin, FSH, TSH, LH, GH, and P53. Ki-67 proliferative index positivity was approximately 1%.

## Treatment

The patient was treated with pasireotide, a second-generation somatostatin analog with high affinity for SSTRs 1, 3, and 5. An initial dose of 40 mg monthly was later increased to 60 mg monthly. Over 6 months, her tumor size stabilized, and her perceived health and quality of life improved significantly.

## Outcome and Follow-up

**Figure 5.**
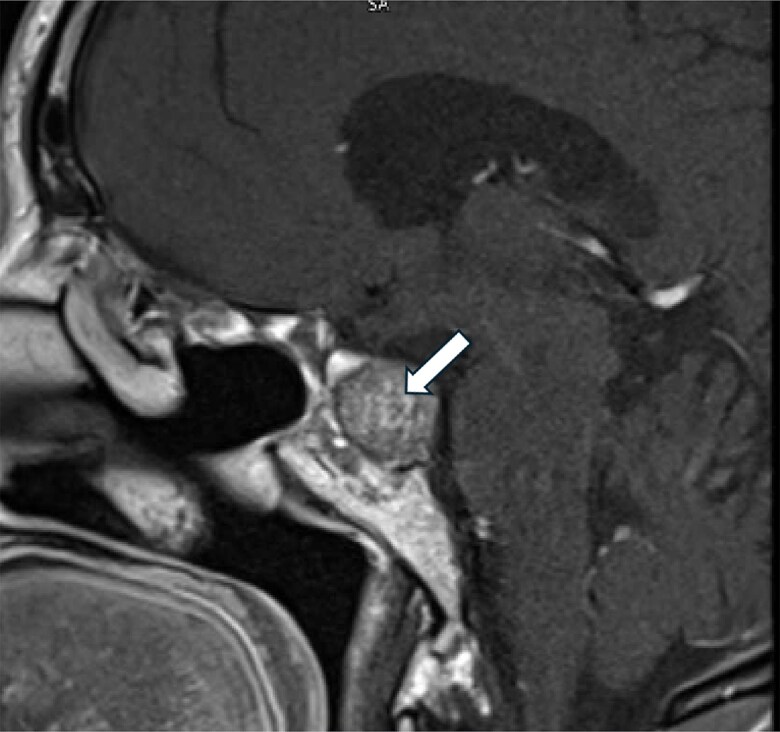
Pituitary macroadenoma measuring 22 × 20 × 17 mm in anterior-posterior, transverse, and craniocaudal dimensions (July 2024).

After 6 months of pasireotide treatment, the patient's tumor size had changed minimally, from 22.1 × 18.7 × 15.8 mm in anterior-posterior, transverse, and craniocaudal dimensions in May 2023 to 22.1 × 18.1 × 15.4 mm in January 2024. Her headaches resolved completely, and she reported significant weight loss and improved well-being. After 1-year follow-up, the tumor size very slightly increased to 22 × 20 × 17 mm in July 2024 (Fig. 5), and the patient continued to experience an improved quality of life. Four months after stopping pasireotide, her HbA1C improved to 9.4%.

## Discussion

This case report illustrates how a patient with an aggressive and treatment-resistant pituitary adenoma responded to pasireotide that stabilized tumor growth and produced a dramatic improvement in tumor-related symptoms, body weight, and her perceived quality of life. Although the role of second-generation SSAs like pasireotide in nonfunctioning pituitary adenoma has not been fully elucidated, there is some evidence to support this treatment approach [5]. Of the 5 SSTRs identified, SSTR2 and SSTR3 are expressed in most nonfunctioning pituitary adenoma samples, whereas SSTR5 is only expressed in a minority of tumors [5]. Given the potent antiproliferative, proapoptotic, and antiangiogenic activities of SSTR3, targeting this receptor with a multireceptor SSA such as pasireotide may be potentially beneficial in treating aggressive nonfunctioning pituitary adenoma [12, 13].

In clinical studies of patients with nonfunctioning pituitary adenoma, octreotide had little effect on tumor mass reduction but was associated with stabilization of postsurgical tumor remnants; in these patients, the SSTR5 subtype was the most abundant in the tumor, followed by SSTR3 [6]. Overall, in clinical studies exploring the efficacy of octreotide or lanreotide in nonfunctioning pituitary adenoma, tumor size reduction was achieved in 12%, an increase was reported in 5%, and no change was reported in 83% of patients receiving these SSAs [14]. Although the percentage of patients who experienced an increase in tumor size during treatment was only 5%, the follow-up was generally too short to draw a conclusion on the potential benefits of octreotide in preventing tumor regrowth [14]. Octreotide has a high affinity for SSTR2 and inhibits the proliferation of the cells expressing the *sstr2* gene by activating the tyrosine phosphatase pathway. Pasireotide binds with a higher affinity to SSTR1 (30-fold), SSTR3 (5-fold), and SSTR5 (39-fold), and with a slightly lower affinity to SSTR2 compared with octreotide [15]. However, surprisingly, although nonfunctioning pituitary adenomas preferentially express *sst3*, both octreotide and pasireotide increased cell viability. This suggests that there are additional factors besides expression of a particular SSTR that affects the response to SSA [16]. Moreover, in vitro data have shown that pasireotide can inhibit nonfunctioning pituitary adenoma cell viability by also inhibiting vascular endothelial growth factor secretion [17]. In a head-to-head comparison of octreotide and pasireotide in a rat model of spontaneous nonfunctioning pituitary adenoma, pasireotide showed a superior antitumor effect compared to octreotide, particularly in female rats, which also expressed more SSTR3 than males [18].

A phase 2 clinical study of 20 patients with clinically nonfunctioning pituitary adenoma receiving 60 mg pasireotide LAR (PASSION I) found that pasireotide could stabilize tumor growth and improve symptoms, reducing tumor size by at least 20% in 16.7% of patients [8, 15].

Pasireotide has previously demonstrated promising results in alleviating headaches associated with pituitary tumors, particularly in patients with acromegaly [19, 20]. This effect is likely mediated through somatostatin receptors that modulate analgesic and anti-inflammatory responses [19]. However, other mechanisms may well be contributing, as we have observed this in several patients who are taking pasireotide (unpublished observation). Pasireotide appears to be a promising option for managing headaches in patients with pituitary adenomas, especially in cases where other treatments have failed to provide adequate relief.

The weight loss observed in the patient could be due to multiple factors, including nausea and diarrhea; although uncontrolled diabetes could also contribute to this weight loss, it was probably more related to being more physically active, since the patient’s general condition improved with pasireotide.

In light of both the pharmacological rationale for exploring pasireotide and adding to previous reports on stabilization of tumor growth and improvement in symptoms with SSAs, the current report opens a new possibility for exploring the efficacy of pasireotide for the treatment of nonfunctioning pituitary adenoma.

Like the other pituitary tumors, the expression of somatostatin receptor ligands in nonfunctioning pituitary adenoma is heterogenous, with SSTR3 being the abundant subtype in these tumors [12]. The lack of significant effect of the current first- and second-generation SSAs on nonfunctioning pituitary adenoma may await the development of specific SST3 analogs [21]. Another treatment option for patients with invasive nonfunctioning pituitary adenoma is the oral nonalkylating agent temozolamide, which appears to be promising in some patients. However, its response appears to be lower in nonfunctioning pituitary adenoma vs functioning pituitary adenomas. Halevy et al summarized a case series totaling 100 patients with pituitary tumors; 27 of them had nonfunctioning adenoma. The response rate to temozolamide in this latter subset was 22%, and 48% had stable disease [22]. However, the recurrence rate can be up to 50%, as was shown by Hirohata et al [23]. Recently Lamas et al showed a not dissimilar response rate; however, coadministration of radiotherapy may improve progression-free survival [24]. The use of nuclear medicine techniques may also offer some options to treat resistant nonfunctioning pituitary adenoma via targeting the expressed somatostatin receptors. Several reports suggest that the use of 177-lutetium DOTATOC in peptide receptor radionuclide therapy may offer some hope in selected cases [25-27].

This case report highlights short-term tumor size control and significant clinical improvement after initiating medical treatment with pasireotide in a patient with an invasive nonfunctioning pituitary adenoma resistant to conventional treatment approaches for many years. The marked improvement in the patient's symptoms and perceived daily quality of life underscores the potential of pasireotide as a therapeutic option for patients with resistant nonfunctioning pituitary adenoma. This case opens new horizons for the use of pasireotide in treating challenging pituitary tumors, warranting further investigation into its efficacy and long-term benefits.

## Learning Points

- Pasireotide may stabilize tumor size in aggressive, treatment-resistant nonfunctioning pituitary adenoma.
- This treatment option can improve symptoms such as headaches and visual disturbances.
- The multireceptor targeting of pasireotide may be particularly effective in nonfunctioning pituitary adenoma expressing different SSTRs
- Further investigation into pasireotide's role in managing nonfunctioning pituitary adenoma is warranted.

## Data Availability

Original data generated and analyzed during this study are included in this published article.
